# Does Ambroxol alleviate kidney ischemia-reperfusion injury in rats?

**DOI:** 10.22038/IJBMS.2022.64330.14148

**Published:** 2022-08

**Authors:** Çağrı Gültekin, Serkan Sayiner, Şule Çetinel, Ahmet Özer Şehirli

**Affiliations:** 1 Department of Surgery, Faculty of Veterinary Medicine, Near East University, 99138, Nicosia, Cyprus; 2 Department of Biochemistry, Faculty of Veterinary Medicine, Near East University, 99138, Nicosia, Cyprus; 3 Department of Histology and Embryology, School of Medicine, Marmara University, 34722 Istanbul, Turkey; 4 Department of Pharmacology, Faculty of Dentistry, Near East University, 99138, Nicosia, Cyprus

**Keywords:** Ambroxol, Distant organ effect, Kidney ischemia, reperfusion, Pro-inflammatory cytokines, Rat

## Abstract

**Objective(s):**

Ischemia-reperfusion injury is a life-threatening clinical problem that can occur after transplantation or a number of clinical procedures. The purpose of the study was to investigate the effects of Ambroxol on kidney damage caused by experimentally induced ischemia-reperfusion injury in rats.

**Materials and Methods:**

Wistar albino rats were divided into 3 groups: Control (CTR, n=6), Kidney ischemia-reperfusion (K-IR, n=6), And kidney ischemia reperfusion+Ambroxol (K-IR+AMB, n=6). In K-IR+AMB group, Ambroxol (30 mg/kg) was administered orally 30 min before the ischemia period. K-IR and K-IR+AMB groups underwent 45 min of kidney ischemia followed by a 6-hour reperfusion period. At the end of the reperfusion period, blood and kidney tissue samples were collected after euthanasia. From the blood samples, BUN and creatinine levels were determined to assess kidney function, and TNF-α and IL-1β concentrations were evaluated to determine inflammatory response.

**Results:**

While serum BUN, creatinine activities, and TNF-α and IL-1β concentrations were higher in both IR groups compared with the CTR group, these values were found to be lower in the K-IR+AMB group compared with the K-IR group. Histopathological examination revealed that interstitial edema and desquamation of tubular cells in the K-IR group were more severe than in the K-IR+AMB group.

**Conclusion:**

Ambroxol treatment alleviated the production of pro-inflammatory cytokines and the harmful cellular effects in the tubular cells.

## Introduction

Ischemia-Reperfusion (IR) is defined as the damage caused by the temporary interruption of blood flow to tissues or an organ, following the restoration of blood flow (1). IR injury is a crucial clinical and surgical problem affecting many different organs such as the kidney, brain, heart, liver, lung, and intestine (2-4). Kidney IR injury can be caused by sepsis, shock, hydronephrosis, open renal stone surgery, partial nephrectomy, transplantation, bleeding, and resuscitation (5). Tumor necrosis factor-alfa (TNF-α) is a critical pro-inflammatory cytokine in kidney IR (K-IR) injury. Excessive production of TNF-α causes a variety of damage to kidney cells, including cell apoptosis, glomerular endothelial damage, fibrin deposition, cellular infiltration, and renal failure (6). After K-IR injury, the renal tubular epithelium produces pro-inflammatory cytokines such as interleukin-1β (IL-1β) which potentiate inflammation (7, 8).

One of the strategies to reduce IR damage involves the administration of pharmacological agents. Many antioxidant agents that act through different mechanisms have been tested clinically and experimentally against K-IR (9). Ambroxol (2-amino-3,5-dibromo-N-[trans-4-hydroxycyclohexyl] benzylamine) is a drug used in respiratory tract diseases due to its mucolytic and secretory properties (10). The effectiveness of Ambroxol on pro and anti-inflammatory cytokines has previously been demonstrated (11, 12). Ambroxol reduces lipopolysaccharide-induced cytokine synthesis in macrophages, attenuates LPS-stimulated superoxide anion and hydrogen peroxide production, and reduces LPS-induced nitric oxide production. It has been reported that the anti-inflammatory effect of Ambroxol reduces TNF-α and neutrophil infiltration in the lungs of rats (13). In addition, Ambroxol also reduces the release or production of cytokines such as IL-1β (14). 

Renal ischemia-reperfusion damage, which can occur for a variety of reasons, is a common cause of acute renal failure with a high mortality risk due to elevated cytokine production. Therefore, investigations including the testing of new therapeutic medicines in treatment protocols are critical. In this study, the effects of Ambroxol, with known anti-inflammatory properties, on pro-inflammatory cytokines were used in a rat kidney ischemia-reperfusion damage model to investigate its efficacy in the treatment of renal ischemia-reperfusion injury.

## Materials and Methods

***Ethical statement***

Approval for the study protocol was obtained from the local animal ethics committee of Near East University (Protocol no: 2019-97). The laboratory staff was blinded to the groups and the administration protocols allocated to the rats. The number of animals used in the experiment was determined to use the least number of animals that would provide statistically significant results.

***Animals***

Eighteen outbred Wistar albino rats, with a weight between 200 and 250 g, including both sexes, were enrolled in the study. All rats were housed in a controlled environment, (humidity (60%), temperature-controlled (22 ± 2 °C) with a 12-hr light/dark cycle ), using conventional cages (solid plastic, rectangular shaped, 20 cm high with a wire mesh lid). Pelleted rat food and water were provided *ad libitum*.

***Experimental model***

Rats were divided into three groups: control group (CTR, n=6) (rats operated without treatment), kidney ischemia-reperfusion group (K-IR, n=6), and kidney ischemia/reperfusion + Ambroxol group (K-IR+AMB, n=6). Exclusion criteria were determined as rats dying during ischemia and/or reperfusion period. No rats were excluded from this study. A liquid form of Ambroxol was obtained from Bilim İlaç San.ve Tic. A.Ş., Turkey and was administered at 30 mg/kg orally in K-IR and K-IR+AMB groups 30 mins before anesthesia (15). All groups were anesthetized by intraperitoneal injection using a mixture of Ketamine (10% Ketamine, Dutchfarm ®, 100 mg/kg) and Xylazine (2% Vetaxyl, Vetagro ®, 10 mg/kg) and fixed in a supine position. Access to the abdominal cavity was performed via a ventral midline incision. In the K-IR and K-IR+AMB groups, the right kidneys were removed. The renal artery and veins of the left kidney were ligated for 45 min to induce ischemia until a color change in the left kidney was observed. The kidneys were reperfused for 6 hr by opening the ligature (16). At the end of the reperfusion period, the rats were euthanized with an overdose of Xylazine+Ketamine anesthesia. The left kidneys were excised, and blood samples were collected into serum separator tubes. All procedures were performed by the same veterinary surgeon in the experimental working unit under the conditions of the housing room.

***Biochemical analysis***

Blood samples were centrifuged at 1500 g x 10 min following coagulation. Then, sera were separated and kept at a temperature of -20 °C until analyzed. Alkaline phosphatase (ALP, U/l), lactate dehydrogenase (LDH, U/l) enzyme activities, blood urea nitrogen (BUN, mg/dl), and creatinine (mg/dl) levels were quantified to assess renal function. Assays were performed using an automated analyzer (BS120, Mindray, Shenzhen, China) and ready-to-use commercial test kits. 

Concentrations of TNF-α (pg/ml) and IL-1β (pg/ml) in sera were determined by rat-specific ELISA assay kits (Rat TNF-α ELISA Kit Catalog No: E-EL-R0019; Rat IL-1β Catalog No: E-EL-R001, Elabscience Biotechnology Inc., TX, USA). Assays were carried out following the manufacturer’s directions.

***Histopathological analysis***

For light microscopic investigations, the removed kidneys were fixed in 10% neutral-buffered formalin solution, dehydrated in alcohol concentration series, cleared in toluene, and embedded in paraffin blocks. The kidney sample paraffin blocks were serially sectioned at an average thickness of 5 µm. Histopathological analyses were conducted on hematoxylin-eosin-stained samples using the bright field mode of a light microscope (Zeiss-Axio Scope A1, Carl Zeiss, Gottingen, Germany).

*Statistical Analysis*

Statistical analyses were carried out using GraphPad Prism 9.1.2 (GraphPad Software, San Diego, CA, USA). All results are expressed as the means ± 1SD. One-way analysis of variance (ANOVA) was used to compare quantified parameters between experimental groups. Tukey’s test was used for further analysis of binary comparisons. *P*-values below 0.05 were regarded as significant.

## Results

ALP and LDH activities (Table 1) were measured as mediators of kidney injury to assess the effects of IR injury on renal cells, as well as the reducing effect of Ambroxol. After the IR injury, a significant elevation in ALP and LDH activities in the K-IR group was detected in comparison with the CTR group (respectively, *P*<0.01 and *P*<0.001). However, in the Ambroxol administrated group (K-IR+AMB) ALP and LDH activities significantly decreased compared with the K-IR group (*P*<0.05). 

BUN and creatinine parameters were quantified as indirect indicators of glomerular filtration rates, thus addressing kidney function (Table 1). The IR injury provoked rising BUN and creatinine levels in the K-IR group compared with the CTR group (both *P*<0.0001). However, Ambroxol administration (K-IR+AMB) significantly decreased BUN and creatinine levels compared with the K-IR group (both *P*<0.01). 

**Figure 1:**
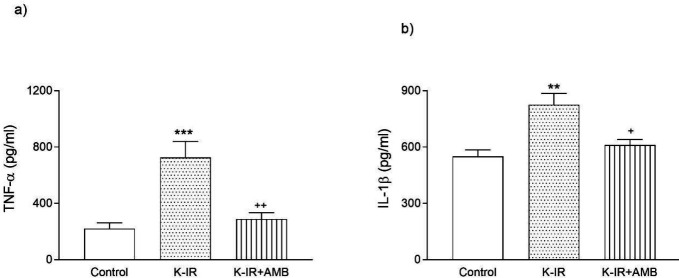
Plasma TNF-α (a) and IL-1β (b) activities in the control (CTR), kidney ischemia/reperfusion (K-IR), and kidney ischemia/reperfusion +Ambroxol (K-IR+AMB) groups in rats. ** *P*<0.01 and *** *P*<0.001, compared with the control (CTR) group. + *P*<0.05 and ++ *P*<0.01, compared with the K-IR group of rats

The pro-inflammatory markers, TNF-α and IL-1β, were measured to assess inflammatory response against IR injury and the effect of Ambroxol administration. Rats in the K-IR group secreted significantly higher levels of TNF-α (*P*<0.001) and IL-1β (*P*<0.01) than rats in the CTR group. Nevertheless, TNF-α and IL-1β levels decreased in the K-IR+AMB group compared with the K-IR group, *P*<0.01 and *P*<0.05, respectively (Figure 1).

**Figure 2:**
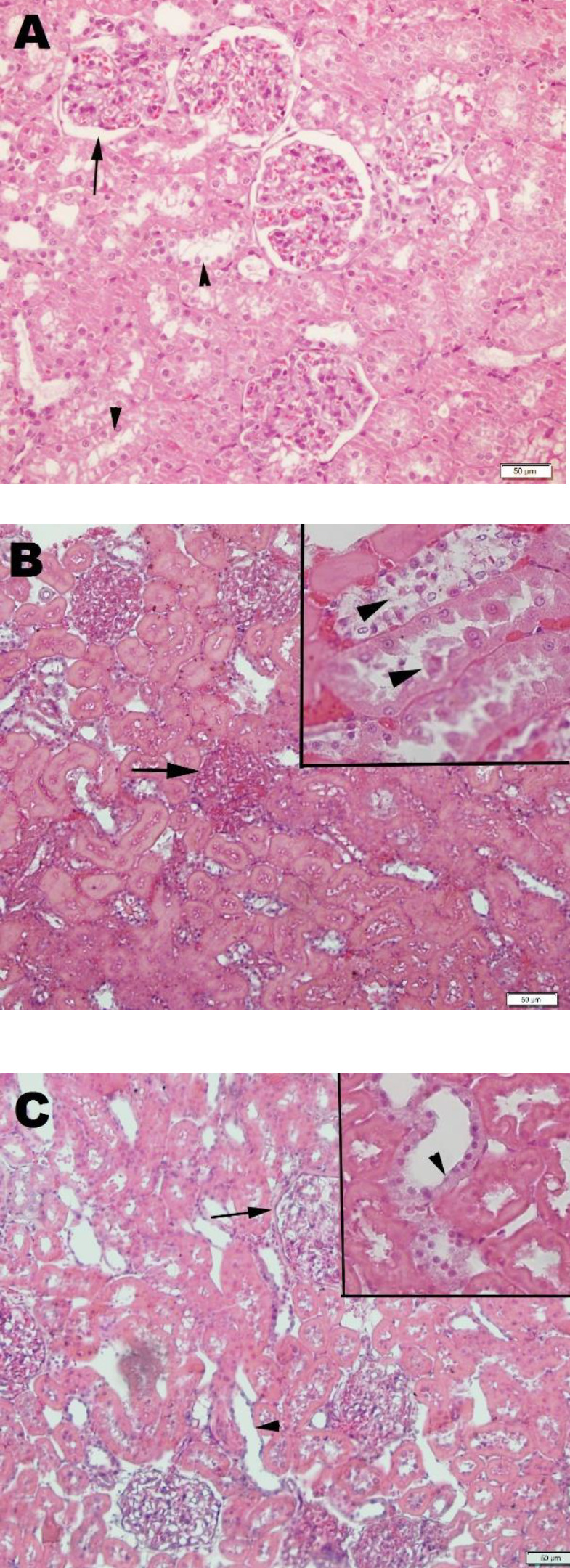
(a) Regular morphology in kidney glomerulus (arrow) and tubuli (arrowheads) of rats in the control group. (b)In the rats of the ischemia-reperfusion group, heavy interstitial edema which constricts glomeruli (arrow), dense desquamation of tubular cells (arrowheads). (c) In the rats of the ischemia-reperfusion and Ambroxol group, regression of interstitial edema (arrow) and re-establishment of tubular cells (arrowheads). HE staining, inset (20 μm)

The histopathological findings show that the kidney tissue exhibited regular glomeruli and tubuli in the CTR group (Figure 2a), whereas the K-IR and K-IR+AMB groups had dilated tubuli, edema in the interstitium, and desquamated tubular cells (Figure 2b). On the other hand, interstitial edema and desquamation of tubular cells were found to be decreased in the K-IR+AMB group (Figure 2c).

**Table 1:** Plasma BUN, creatinine, ALP, and LDH activities in the control (CTR), kidney ischemia/reperfusion (K-IR), and kidney ischemia/reperfusion +Ambroxol (K-IR+AMB) groups of rats

	**CTR**	**K-IR**	**K-IR+AMB**
**ALP (U/L)**	91.4 ± 3.3	125.2 ± 7.4 **	100.7 ± 4.6 ^+^
**LDH (U/L)**	3050 ± 463	5987 ± 456 ***	4313 ± 212 ^+^
**BUN (mg/dl)**	21.69 ± 0.81	32.25 ± 1.54 ****	25.78 ± 0.66 ^++^
**creatinine (mg/dl)**	0.68 ± 0.02	1.18 ± 0.04 ****	0.94 ± 0.04 ***^, ++^

** *P*<0.01; *** *P*<0.001 ; and **** *P*<0.0001, compared with the control (CTR) group. + *P*<0.05 and ++ *P*<0.01, compared with the K-IR group

BUN: Blood urea nitrogen; ALP: Alkaline phosphatase; LDH: Lactate dehydrogenase

## Discussion

In the pathophysiological mechanisms of IR injury, many factors play an important role, such as cytokine formation, cellular damage, and inflammation, involving complex signaling pathways. Although there are many different approaches to the use of drugs against IR injury, the main goal in the management of IR injury is to protect cells or minimize cellular damage (17). Therefore, we investigated the effects of Ambroxol on kidney damage caused by IR injury in this study.

BUN and creatinine are metabolites excreted by glomerular filtration, and their plasma concentrations increase in cases where glomerular filtration is affected, such as acute renal failure, intoxication, and kidney transplant rejection (18). K-IR injury causes tubular and glomerular dysfunction, especially during the reperfusion phase, such as tubular cell necrosis and damage, glomerular damage, and decreased glomerular filtration rate (16, 19). In this study, BUN and creatinine levels were measured to detect kidney damage due to glomerular and tubular damage. According to BUN, creatinine levels, and histopathological examination results we demonstrated that ischemia-reperfusion injury impaired glomerular and tubular structures and functions in both groups. Although this deterioration was observed to be more severe in the K-IR group, it was less severe in the Ambroxol group, indicating that Ambroxol is effective in preserving renal tubular and glomerular structures.

In K-IR injury, the release of inflammatory cytokines such as IL-1β, which induces the mobilization of leukocytes and determines inflammatory activation, and TNF-α, which provides neutrophil infiltration, increases kidney injury and contributes to inflammation in tubular cells (1, 20, 21). In some studies, it has been shown that Ambroxol affects inflammatory cytokines and reduces TNF-α and IL-1β levels (13, 14). In this study, the levels of inflammatory cytokines TNF-α and IL-1β were measured to determine K-IR injury. Our results show that Ambroxol reduced the harmful effects of K-IR injury on kidney tissue by decreasing the levels of TNF-α and IL-1β, which are essential inflammatory cytokines in K-IR injury. In studies on cytokine expression, Ambroxol has been shown to reduce TNF-α and IL-1β levels, especially in inflammation models (22, 23). However, the effects of Ambroxol on K-IR injury have not been evaluated to date. This study demonstrated the protective effect of Ambroxol against the harmful effects of pro-inflammatory cytokines TNF-α and IL-1β after kidney ischemia-reperfusion injury.

In acute renal failure, multiple organ failure and secondary diseases can often be seen and concomitant diseases usually determine the severity of the condition. Furthermore, elevated levels of pro-inflammatory cytokines and K-IR damage in the liver have been shown to raise serum LDH and ALP activities (24-28) Besides, recent research suggests that the activity of ALP and LDH enzymes is also a good marker for detecting renal cellular damage in acute kidney injury (29-31). Thus, LDH and ALP activities were considered to assess the effects of K-IR injury on the liver and kidney. LDH and ALP values in the K-IR group were higher than those in the K-IR+AMB group, similar to prior findings. According to these results, Ambroxol protects against K-IR injury both directly by lowering cytokine levels and indirectly by lowering LDH and ALP levels on kidney cells or liver cells, which provide compensation in the body against cytokines generated after K-IR injury.

## Conclusion

In this study, we investigated the effects of Ambroxol on TNF-α and IL-1β, which cause histopathological changes, after experimentally induced K-IR injury. Our findings were that Ambroxol reduced the release of TNF-α and IL-1β, thus reducing the effects of IR on the kidney. The effects of Ambroxol at different doses and on other pathophysiological mechanisms that occur with ischemia-reperfusion are subjects that need to be investigated, and our future studies aim to investigate these issues. In conclusion, in line with the findings obtained from the study, Ambroxol can be used as one of the preventive, therapeutic approaches to the effects of K-IR injury.

## Authors’ Contributions

ÇG, AÖŞ, and SS Provided study conception and design; ÇG and AÖŞ Performed experiments; SS, ŞÇ, and AÖŞ Analyzed data; ÇG, AÖŞ, and SS Interpreted the results and wrote the manuscript.

## Funding

The authors received no financial support for the research, authorship, and/or publication of this article.

## Data Statement

The data presented in this study are available via the corresponding author’s e-mail.

## Conflicts of Interest

The authors declared no potential conflicts of interest with respect to the research, authorship, and/or publication of this article.
